# Crystal structure refinement of magnesium zinc divanadate, MgZnV_2_O_7_, from powder X-ray diffraction data

**DOI:** 10.1107/S2056989021004503

**Published:** 2021-05-07

**Authors:** Stephanie J. Hong, Jun Li, Mas A. Subramanian

**Affiliations:** a Department of Chemistry, Oregon State University, Corvallis, Oregon 97331, USA

**Keywords:** crystal structure, powder X-ray diffraction, magnesium zinc divanadate, MgZnV_2_O_7_, thortveitite-related structures

## Abstract

The crystal structure of magnesium zinc divanadate, MgZnV_2_O_7_, was determined and refined from laboratory X-ray powder diffraction data. The title compound was synthesized by a solid-state reaction at 1023 K in air. The crystal structure is isotypic with Mn_0.6_Zn_1.4_V_2_O_7_ (*C2*/*m*; *Z* = 6) and is related to the crystal structure of thortveitite. The asymmetric unit contains two metal sites with statistically distributed magnesium and zinc atoms with the atomic ratio close to 1:1. One (Mg/Zn) metal site (*M*1) is located on Wyckoff position 8*j* and the other (*M*2) on 4*h*. Three V sites (all on 4*i*), and eight O (three 8*j*, four 4*i*, and one 2*b*) sites complete the asymmetric unit. The structure is an alternate stacking of V_2_O_7_ layers and (Mg/Zn) atom layers along [20]. It is distinct from other related structures in that each V_2_O_7_ layer consists of two groups: a V_2_O_7_ dimer and a V_4_O_14_ tetra­mer. Mixed-occupied *M*1 and *M*2 are coordinated by oxygen atoms in distorted trigonal bipyramidal and octa­hedral sites, respectively.

## Abstract 2

The crystal structure of magnesium zinc divanadate can be described by an alternate stacking of V_2_O_7_ layers and (Mg/Zn) atom layers. Each V_2_O_7_ layer consists of a V_2_O_7_ dimer and a V_4_O_14_ tetra­mer.

## Chemical context   

**Figure 1:**
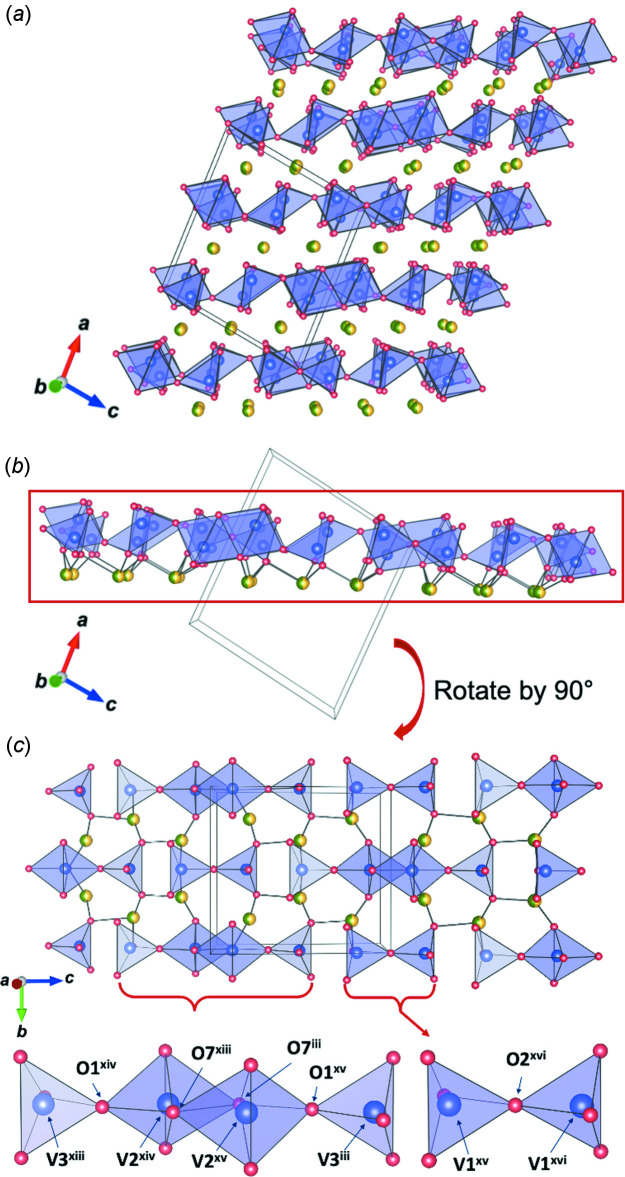
The crystal structure of MgZnV_2_O_7_ with VO_4_ tetra­hedra, VO_5_ trigonal bipyramids (light purple), and (Mg/Zn) atoms (green/yellow). (*a*) overview of the structure, (*b*) a selected slab of one V_2_O_7_ layer and the adjacent (Mg/Zn) layer, and (*c*) a top view of the slab of (*b*) and magnified local structure of V_4_O_14_ tetra­meric and V_2_O_7_ dimeric units. [Symmetry codes: (xiii) −*x* + 1, −*y* + 1, −*z*; (xiv) *x*, *y* + 1, *z* − 1; (xv) −*x* + 1, −*y* + 1, −*z* + 1; (xvi) *x* + 1, *y* + 1, *z* + 1.]

Mixed vanadium oxides with tetra­hedrally coordinated penta­valent vanadium ions have been used as catalysts in the heterogeneous oxidation process (Chang & Wang, 1988 ▸). Since there is a strong correlation between the crystal structure and its properties, the phase relations of vanadates have been thoroughly investigated. During the course of studying the phase diagram in the MgO–ZnO–V_2_O_5_ system, a new phase was identified by its X-ray diffraction pattern in the solid-solution range between (Mg_0.80_Zn_1.20_)V_2_O_7_ and (Mg_1.16_Zn_0.84_)V_2_O_7_, which was completely different from Mg_2_V_2_O_7_ or Zn_2_V_2_O_7_ (Chang & Wang, 1988 ▸). The crystal structure of the new phase has not been reported to date. We present here the crystal structure of MgZnV_2_O_7_ (Fig. 1 ▸), as determined and refined from laboratory powder X-ray diffraction data (Table 1 ▸).

## Structural commentary   

The crystal structure of magnesium zinc divanadate, MgZnV_2_O_7_, is isotypic with Mn_0.6_Zn_1.4_V_2_O_7_ (Knowles *et al.*, 2009 ▸), where statistically distributed Mg and Zn atoms (Mn and Zn for Mn_0.6_Zn_1.4_V_2_O_7_) are located in disordered environments in the crystal structure. The unit-cell volume of MgZnV_2_O_7_ is smaller than that of Mn_0.6_Zn_1.4_V_2_O_7_ by 1.65%.

The crystal structure of MgZnV_2_O_7_ is shown in Fig. 1 ▸ *a*. There are (Mg1/Zn1) (on Wyckoff position 8*j*, site symmetry 1), (Mg2/Zn2) (on 4*h*, 2), three V (all on 4*i*, *m*), and eight O (three 8*j*, four 4*i*, and one 2*b*, 2/*m*) sites in the asymmetric unit, where (Mg1/Zn1) and (Mg2/Zn2) represent statistically distributed magnesium and zinc atoms with the atomic ratio close to 1:1.

The crystal structure can be described as an alternate stacking of V_2_O_7_ layers and (Mg/Zn) atom layers along [20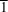] (Fig. 1 ▸ *b*). Each V_2_O_7_ layer consists of two groups: a V_2_O_7_ dimer and a V_4_O_14_ tetra­mer. For illustration, a slab of one V_2_O_7_ layer and the adjacent (Mg/Zn) layer is shown in Fig. 1 ▸ *c*, which is rotated by 90° from Fig. 1 ▸ *b*. Two corner-sharing (V1)O_4_ tetra­hedra form the dimeric group. Two (V3)O_4_ tetra­hedra and two (V2)O_5_ trigonal bipyramids form the tetra­meric group, with a sequence of (V3)O_4_–(V2)O_5_–(V2)O_5_–(V3)O_4_. The two trigonal bipyramidal units in the middle are edge-sharing, each of which is corner-sharing with the adjacent terminal tetra­hedron. (Mg1/Zn1) and (Mg2/Zn2) are coordinated by oxygen atoms in a distorted trigonal bipyramidal and a distorted octa­hedral environment, respectively (Table 2 ▸).

**Figure 2:**
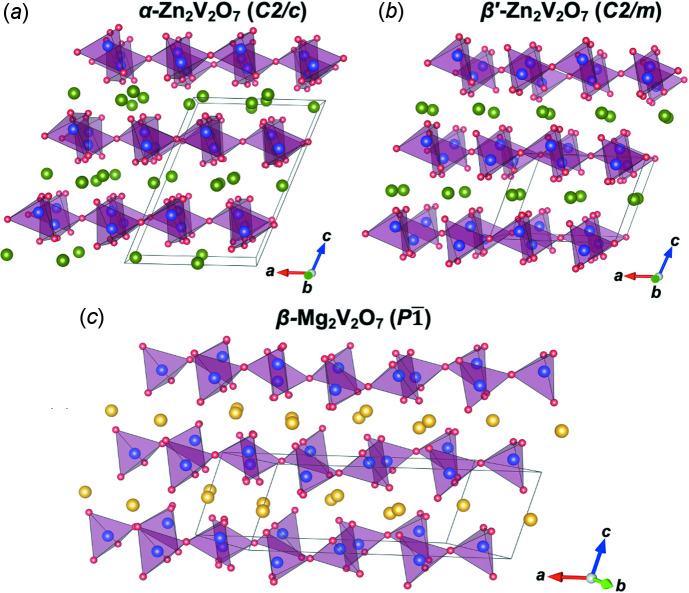
Crystal structure of (*a*) *α*-Zn_2_V_2_O_7_, (*b*) thortveite-type *β*’-Zn_2_V_2_O_7_, and (*c*) *β*-Mg_2_V_2_O_7_.

The MgZnV_2_O_7_ structure (*C2*/*m*, *Z* = 6) is closely related to thortveitite-type *α*-Zn_2_V_2_O_7_ (*C2*/*c*, *Z* = 4) (Gopal & Calvo, 1973 ▸), thortveite-type *β*’-Zn_2_V_2_O_7_ (*C2*/*m*, *Z* = 2) (Krasnenko *et al.*, 2003 ▸), and *β*-Mg_2_V_2_O_7_ (*P* 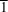, *Z* = 2) (Gopal & Calvo, 1974 ▸), as shown in Fig. 2 ▸, in which they have an alternate stacking of V_2_O_7_ layer and Zn or Mg layers. However, in contrast to MgZnV_2_O_7_, they only contain the V_2_O_7_ dimer groups. The relationships between other thortveitite-related phases are also well described in a previous work (Knowles *et al.*, 2018 ▸).

To check the refined structure model, empirical bond-valence sums (BVSs) were calculated (Brown & Altermatt, 1985 ▸; Brese & O’Keeffe, 1991 ▸), with the program *Valence* (Hormillosa *et al.*, 1993 ▸). The expected charges of the ions match the obtained BVS values (given in valence units): (Mg1/Zn1) = 1.96, (Mg2/Zn2) = 2.11, V1 = 6.08, V2 = 4.31, V3 = 4.69, O1 = 1.60, O2 = 2.26, O3 = 2.25, O4 = 1.71, O5 = 2.10, O6 = 1.87, O7 = 2.02, and O8 = 2.38. The high value for V1 comes from the relatively short V—O distances (Table 2 ▸). The restrained distance was slightly longer than the final values, however, the refinement led to the shorter distances. Short bond lengths (1.56–1.60 Å) were also found in other materials, such as BiBa_2_(VO_4_)(V_2_O_7_) (Huang *et al.*, 1994 ▸) Mg_2_(V_2_O_7_) (Nielsen *et al.*, 2001 ▸) or Th(V_2_O_7_) (Launay *et al.*, 1992 ▸). The final atomic positions were confirmed in the Fourier maps (observed and difference map).

## Synthesis and crystallization   

MgZnV_2_O_7_ was synthesized by a solid-state reaction from a mixture of Mg(CH_3_COO)_2_·4H_2_O (98.0–102.0%, Alfa-Aesar), ZnO (99.99%, Aldrich) and V_2_O_5_ (99.99%, Aldrich) with a nominal composition of Mg:Zn:V = 1:1:2. The mixture was thoroughly ground in an agate mortar with acetone, dried, pressed into a pellet, heated in air at 673 K for 3 h, at 943 K for 6 h, and again at 1023 K for 6 h with inter­mediate grinding and pressing. For the powder X-ray diffraction measurement, the pellet was ground again in an agate mortar and the resultant powder was dispersed on a zero-background Si sample holder.

## Refinement details   

**Figure 3:**
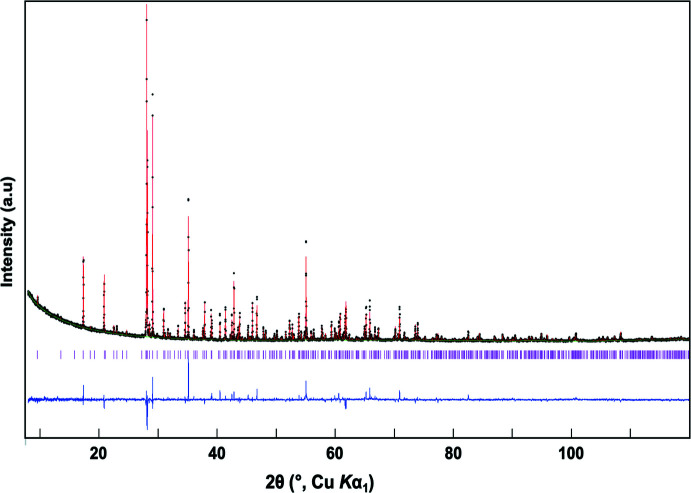
Powder X-ray diffraction Rietveld refinement profiles for MgZnV_2_O_7_ from room-temperature data. Black dots mark experimental data, the solid red line represents the calculated profile, and the solid green line is the background. The bottom trace presents the difference curve (blue) and the ticks denote the expected Bragg reflection positions (magenta).

Details of the crystal data collection and structure refinement are summarized in Table 1 ▸ and the supporting information. Powder X-ray diffraction (PXRD) data for MgZnV_2_O_7_ were collected from a Bragg-Brentano diffractometer (PANalytical, 2011 ▸) using Cu *K*α_1_ radiation, a focusing primary Ge(111) monochromator (λ = 1.5405 Å) and a position-sensitive PIXcel 3D 2×2 detector. The angular range was set to 8°≤ 2θ ≤ 120°, with a step of 0.0131° and a total measurement time of 8 h at room temperature. The PXRD pattern was indexed using the *DICVOL* algorithm (Boultif & Louër, 2004 ▸) run in *WINPLOT* (Roisnel & Rodríguez-Carvajal, 2000 ▸) through the positions of 26 reflections, resulting in a monoclinic unit cell (step 1). The space groups from the systematic reflection conditions were suggested to be *C2*/*m*, *C2*, or *Cm*, which were indistinguishable from the reflection conditions. The highest symmetry, *C2*/*m*, was chosen first to determine the structure (step 2), and confirmed later. All the reflections were well indexed, except for a few minor unidentified impurity peaks. The structure determination was performed by a combination of the powder profile refinement program *GSAS* (Larson & Von Dreele, 2000 ▸) and the single-crystal structure-refinement program *CRYSTALS* (Betteridge *et al.*, 2003 ▸). The software *MCE* was used to visualize the three-dimensional Fourier electron-density maps, (Rohlíček & Hušák, 2007 ▸). Initially, a structural model was used with only one dummy atom placed at the (0,0,0) position in the unit cell. A Le Bail fit was used to extract the structure factors from the powder data in *GSAS* (step 3), followed by applying direct methods to build the initial structural solution, using *SHELXS97* (Sheldrick, 2008 ▸) run in *CRYSTALS*, which yielded three vanadium sites as the initial structural model (step 4). The initial dummy atom model was then replaced with the partial model containing only three vanadium atoms, and the Le Bail fit was applied in *GSAS* (step 5). Improved structure factors were then extracted, which were used for the refinement in *CRYSTALS* (step 6). This process (step 5 to 6) was repeated until a complete and satisfactory structural model was obtained. Finally, Rietveld refinement in *GSAS* was employed to complete the structure model, resulting in reasonable isotropic displacement parameters and agreement indices (step 7). The refinement parameters were scale factors, background, unit-cell parameters, peak profile coefficients, atomic coordinates, occupancies for the two (Mg/Zn) sites, common *U*_iso_ for the metal atoms, common *U*_iso_ for the oxygen atoms, and a March–Dollase preferential orientation coefficient (<111> direction). For the final Rietveld refinement cycles, the Mg—O, Zn—O, and V—O bond lengths were restrained with a tolerance value of 0.01 Å with respect to the distances determined from *CRYSTALS*, which matched reasonably well with the radii sums of Shannon (1976 ▸). Atomic coordinates and labeling were finally adapted from isotypic Mn_0.6_Zn_1.4_V_2_O_7_ (Knowles *et al.*, 2009 ▸). The final Rietveld plot is displayed in Fig. 3 ▸.

## Supplementary Material

Crystal structure: contains datablock(s) I. DOI: 10.1107/S2056989021004503/wm5602sup1.cif

CCDC reference: 2080585

Additional supporting information:  crystallographic information; 3D view; checkCIF report

## Figures and Tables

**Table 1:** Experimental details

Crystal data	
Chemical formula	MgZnV_2_O_7_
*M*_r_	303.56
Crystal system, space group	Monoclinic, *C*2/*m*
Temperature (K)	298
*a*, *b*, *c* (Å)	10.32882 (7), 8.50126 (5), 9.30814 (6)
β (°)	98.5748 (5)
*V* (Å^3^)	808.19 (1)
*Z*	6
Radiation type	Cu *K*α_1_, λ = 1.5405 Å
Specimen shape, size (mm)	Irregular, 24.9 × 24.9

Data collection	
Diffractometer	PANalytical Empyrean
Specimen mounting	Dispersed powder
Data collection mode	Reflection
Scan method	Step
2θ values (°)	2θ_min_ = 5.012, 2θ_max_ = 119.991, 2θ_step_ = 0.013

Refinement	
*R* factors and goodness of fit	*R*_p_ = 0.055, *R*_wp_ = 0.076, *R*_exp_ = 0.042, *R*(*F* ^2^) = 0.20886, χ^2^ = 3.276
No. of parameters	40

Computer programs: *X’Pert Data Collector* and *X’Pert HighScore Plus* (PANalytical, 2011 ▸), *GSAS* (Larson & Von Dreele, 2000 ▸), *SHELXS97* (Sheldrick, 2008 ▸), *CRYSTALS* (Betteridge *et al.*, 2003 ▸) and *VESTA* (Momma & Izumi, 2011 ▸).

**Table 2:** Selected bond lengths (Å)

Mg1—O3^i^	1.975 (10)	V1—O3^vii^	1.628 (10)
Mg1—O4^i^	1.929 (9)	V1—O3^viii^	1.628 (10)
Mg1—O5^ii^	2.172 (10)	V1—O8	1.599 (15)
Mg1—O7^iii^	2.081 (11)	V2—O1	1.959 (12)
Mg1—O8^iii^	2.120 (11)	V2—O4	1.745 (9)
Mg2—O3^iv^	2.354 (8)	V2—O4^ix^	1.745 (9)
Mg2—O3^ii^	2.354 (8)	V2—O7^x^	2.032 (11)
Mg2—O5^v^	1.948 (10)	V2—O7^xi^	1.898 (15)
Mg2—O5^i^	1.948 (10)	V3—O1^xi^	1.824 (10)
Mg2—O6^iii^	2.061 (10)	V3—O5^xi^	1.703 (10)
Mg2—O6^vi^	2.061 (10)	V3—O5^xii^	1.703 (10)
V1—O2	1.757 (4)	V3—O6^xi^	1.760 (15)

Symmetry codes: (i) -x+{\script{1\over 2}}, y+{\script{1\over 2}}, -z+1; (ii) x, -y+1, z; (iii) x, y+1, z; (iv) -x, -y+1, -z+1; (v) x-{\script{1\over 2}}, y+{\script{1\over 2}}, z; (vi) -x, y+1, -z+1; (vii) -x, y, -z+1; (viii) -x, -y, -z+1; (ix) x, -y, z; (x) x, y, z+1; (xi) -x+1, y, -z+1; (xii) -x+1, -y, -z+1.

## supplementary crystallographic information

### Crystal data

**Table:**

MgZnV_2_O_7_	*V* = 808.19 (1) Å^3^
*M**_r_* = 303.56	*Z* = 6
Monoclinic, *C*2/*m*	*F*(000) = 864.0
Hall symbol: -C 2y	*D*_x_ = 3.743 Mg m^−^^3^
*a* = 10.32882 (7) Å	Cu *K*α_1_ radiation, λ = 1.5405 Å
*b* = 8.50126 (5) Å	*T* = 298 K
*c* = 9.30814 (6) Å	yellow
β = 98.5748 (5)°	irregular, 24.9 × 24.9 mm

### Data collection

**Table:**

PANalytical Empyrean diffractometer	Data collection mode: reflection
Radiation source: sealed X-ray tube, PANalytical Cu Ceramic X-ray tube	Scan method: step
Specimen mounting: dispersed powder	2θ_min_ = 5.012°, 2θ_max_ = 119.991°, 2θ_step_ = 0.013°

### Refinement

**Table:**

Least-squares matrix: full	Profile function: CW Profile function number 4 with 21 terms Pseudovoigt profile coefficients as parameterized in P. Thompson, D.E. Cox & J.B. Hastings (1987). J. Appl. Cryst.,20,79-83. Asymmetry correction of L.W. Finger, D.E. Cox & A. P. Jephcoat (1994). J. Appl. Cryst.,27,892-900. Microstrain broadening by P.W. Stephens, (1999). J. Appl. Cryst.,32,281-289. #1(GU) = 3.306 #2(GV) = 0.000 #3(GW) = 0.000 #4(GP) = 1.565 #5(LX) = 2.176 #6(ptec) = 0.00 #7(trns) = 0.00 #8(shft) = -0.5558 #9(sfec) = 0.00 #10(S/L) = 0.0005 #11(H/L) = 0.0005 #12(eta) = 0.7500 #13(S400 ) = 0.0E+00 #14(S040 ) = 0.0E+00 #15(S004 ) = 0.0E+00 #16(S220 ) = 0.0E+00 #17(S202 ) = 0.0E+00 #18(S022 ) = 0.0E+00 #19(S301 ) = 0.0E+00 #20(S103 ) = 0.0E+00 #21(S121 ) = 0.0E+00 Peak tails are ignored where the intensity is below 0.0020 times the peak Aniso. broadening axis 0.0 0.0 1.0
*R*_p_ = 0.055	40 parameters
*R*_wp_ = 0.076	0 restraints
*R*_exp_ = 0.042	(Δ/σ)_max_ = 0.03
*R*(*F*^2^) = 0.20886	Background function: GSAS Background function number 1 with 32 terms. Shifted Chebyshev function of 1st kind 1: 684.144 2: -705.826 3: 591.845 4: -383.442 5: 271.953 6: -144.446 7: 80.6035 8: -57.5987 9: 36.6287 10: -27.9916 11: 15.1285 12: -11.9775 13: 12.8819 14: -10.6721 15: 7.66251 16: -8.35018 17: 1.44025 18: -5.00721 19: 5.78817 20: -2.82870 21: 1.93935 22: -1.83947 23: 4.15929 24: 0.506732 25: -0.182215 26: 0.162584 27: 4.98669 28: 0.850932 29: -0.614188 30: -2.46723 31: 3.10631 32: 3.29784
8758 data points	Preferred orientation correction: March-Dollase AXIS 1 Ratio= 0.72861 h= 1.000 k= 1.000 l= 1.000 Prefered orientation correction range: Min= 0.62193, Max= 1.62737
Excluded region(s): The background is too high at low angles and there was no Bragg's peaks.	

### Fractional atomic coordinates and isotropic or equivalent isotropic displacement parameters (Å^2^)

**Table:**

	*x*	*y*	*z*	*U*_iso_*/*U*_eq_	Occ. (<1)
Mg1	0.3477 (3)	0.8175 (3)	0.2035 (3)	0.0069 (3)*	0.508 (4)
Mg2	0.0	0.8191 (5)	0.5	0.0069 (3)*	0.484 (7)
Zn1	0.3477 (3)	0.8175 (3)	0.2035 (3)	0.0069 (3)*	0.492 (4)
Zn2	0.0	0.8191 (5)	0.5	0.0069 (3)*	0.516 (7)
V1	0.0517 (4)	0.0	0.1885 (4)	0.0069 (3)*	
V2	0.3777 (4)	0.0	0.8892 (4)	0.0069 (3)*	
V3	0.6962 (4)	0.0	0.4938 (4)	0.0069 (3)*	
O1	0.4041 (12)	0.0	0.6850 (12)	0.0070 (8)*	
O2	0.0	0.0	0.0	0.0070 (8)*	
O3	0.0123 (9)	0.1586 (10)	0.7538 (9)	0.0070 (8)*	
O4	0.2787 (9)	0.1666 (10)	0.8864 (9)	0.0070 (8)*	
O5	0.3613 (10)	0.1678 (11)	0.4382 (10)	0.0070 (8)*	
O6	0.1338 (13)	0.0	0.5091 (13)	0.0070 (8)*	
O7	0.4380 (14)	0.0	0.1072 (12)	0.0070 (8)*	
O8	0.2081 (15)	0.0	0.2045 (13)	0.0070 (8)*	

### Geometric parameters (Å, º)

**Table:**

Mg1—Mg1^i^	3.104 (6)	V2—O1	1.959 (12)
Mg1—Mg2^ii^	3.185 (3)	V2—O4	1.745 (9)
Mg1—Zn1^i^	3.104 (6)	V2—O4^xv^	1.745 (9)
Mg1—Zn2^ii^	3.185 (3)	V2—O7^xvi^	2.032 (11)
Mg1—V3^iii^	3.307 (5)	V2—O7^xvii^	1.898 (15)
Mg1—O3^iv^	1.975 (10)	V3—Mg1^xviii^	3.307 (5)
Mg1—O4^iv^	1.929 (9)	V3—Mg1^xix^	3.307 (5)
Mg1—O5^v^	2.172 (10)	V3—O1^xvii^	1.824 (10)
Mg1—O7^vi^	2.081 (11)	V3—O5^xvii^	1.703 (10)
Mg1—O8^vi^	2.120 (11)	V3—O5^xx^	1.703 (10)
Mg2—Mg1^ii^	3.185 (3)	V3—O6^xvii^	1.760 (15)
Mg2—Mg1^vii^	3.185 (3)	O1—V2	1.959 (12)
Mg2—Mg2^viii^	3.076 (9)	O1—V3^xvii^	1.824 (10)
Mg2—Zn1^ii^	3.185 (3)	O2—V1	1.757 (4)
Mg2—Zn1^vii^	3.185 (3)	O2—V1^xxi^	1.757 (4)
Mg2—Zn2^viii^	3.076 (9)	O3—Mg1^xxii^	1.975 (10)
Mg2—O3^ix^	2.354 (8)	O3—Mg2^ix^	2.354 (8)
Mg2—O3^v^	2.354 (8)	O3—Zn1^xxii^	1.975 (10)
Mg2—O5^x^	1.948 (10)	O3—Zn2^ix^	2.354 (8)
Mg2—O5^iv^	1.948 (10)	O3—V1^xii^	1.628 (10)
Mg2—O6^vi^	2.061 (10)	O4—Mg1^xxii^	1.929 (9)
Mg2—O6^xi^	2.061 (10)	O4—Zn1^xxii^	1.929 (9)
Zn1—Mg1^i^	3.104 (6)	O4—V2	1.745 (9)
Zn1—Mg2^ii^	3.185 (3)	O5—Mg1^v^	2.172 (10)
Zn1—Zn1^i^	3.104 (6)	O5—Mg2^xxiii^	1.948 (10)
Zn1—O3^iv^	1.975 (10)	O5—Zn1^v^	2.172 (10)
Zn1—O4^iv^	1.929 (9)	O5—Zn2^xxiii^	1.948 (10)
Zn1—O5^v^	2.172 (10)	O5—V3^xvii^	1.703 (10)
Zn1—O7^vi^	2.081 (11)	O6—Mg2^xxiv^	2.061 (10)
Zn1—O8^vi^	2.120 (11)	O6—Mg2^ix^	2.061 (10)
Zn2—Mg1^ii^	3.185 (3)	O6—Zn2^xxiv^	2.061 (10)
Zn2—Mg1^vii^	3.185 (3)	O6—Zn2^ix^	2.061 (10)
Zn2—Mg2^viii^	3.076 (9)	O6—V3^xvii^	1.760 (15)
Zn2—Zn2^viii^	3.076 (9)	O7—Mg1^xxiv^	2.081 (11)
Zn2—O3^ix^	2.354 (8)	O7—Mg1^v^	2.081 (11)
Zn2—O3^v^	2.354 (8)	O7—Zn1^xxiv^	2.081 (11)
Zn2—O5^x^	1.948 (10)	O7—Zn1^v^	2.081 (11)
Zn2—O5^iv^	1.948 (10)	O7—V2^xxv^	2.032 (11)
Zn2—O6^vi^	2.061 (10)	O7—V2^xvii^	1.898 (15)
Zn2—O6^xi^	2.061 (10)	O8—Mg1^xxiv^	2.120 (11)
V1—O2	1.757 (4)	O8—Mg1^v^	2.120 (11)
V1—O3^xii^	1.628 (10)	O8—Zn1^xxiv^	2.120 (11)
V1—O3^xiii^	1.628 (10)	O8—Zn1^v^	2.120 (11)
V1—O8	1.599 (15)	O8—V1	1.599 (15)
V2—V2^xiv^	3.014 (6)		
			
Mg1^i^—Mg1—Mg2^ii^	111.37 (6)	O3^xii^—V1—O3^xiii^	111.9 (8)
Mg1^i^—Mg1—O3^iv^	133.1 (3)	O3^xii^—V1—O8	115.1 (4)
Mg1^i^—Mg1—O4^iv^	131.7 (3)	O3^xiii^—V1—O8	115.1 (4)
Mg1^i^—Mg1—O5^v^	86.7 (2)	O1—V2—O4	98.7 (4)
Mg1^i^—Mg1—O7^vi^	41.8 (3)	O1—V2—O4^xv^	98.7 (4)
Mg1^i^—Mg1—O8^vi^	42.9 (3)	O1—V2—O7^xvi^	154.4 (6)
Mg2^ii^—Mg1—O3^iv^	47.5 (3)	O1—V2—O7^xvii^	74.5 (5)
Mg2^ii^—Mg1—O4^iv^	110.4 (3)	O4—V2—O4^xv^	108.5 (7)
Mg2^ii^—Mg1—O5^v^	36.9 (3)	O4—V2—O7^xvi^	96.1 (4)
Mg2^ii^—Mg1—O7^vi^	116.7 (3)	O4—V2—O7^xvii^	125.7 (3)
Mg2^ii^—Mg1—O8^vi^	120.7 (4)	O4^xv^—V2—O7^xvi^	96.1 (4)
O3^iv^—Mg1—O4^iv^	93.5 (3)	O4^xv^—V2—O7^xvii^	125.7 (3)
O3^iv^—Mg1—O5^v^	84.3 (3)	O7^xvi^—V2—O7^xvii^	79.9 (7)
O3^iv^—Mg1—O7^vi^	103.6 (5)	O1^xvii^—V3—O5^xvii^	99.1 (4)
O3^iv^—Mg1—O8^vi^	167.9 (4)	O1^xvii^—V3—O5^xx^	99.1 (4)
O4^iv^—Mg1—O5^v^	114.4 (4)	O1^xvii^—V3—O6^xvii^	114.7 (7)
O4^iv^—Mg1—O7^vi^	128.8 (4)	O5^xvii^—V3—O5^xx^	113.8 (8)
O4^iv^—Mg1—O8^vi^	94.1 (4)	O5^xvii^—V3—O6^xvii^	114.2 (4)
O5^v^—Mg1—O7^vi^	115.1 (4)	O5^xx^—V3—O6^xvii^	114.2 (4)
O5^v^—Mg1—O8^vi^	84.0 (5)	V2—O1—V3^xvii^	138.0 (7)
O7^vi^—Mg1—O8^vi^	78.8 (4)	V1—O2—V1^xxi^	180.0
Mg1^ii^—Mg2—Mg1^vii^	137.26 (13)	Mg1^xxii^—O3—Mg2^ix^	94.3 (4)
Mg1^ii^—Mg2—Mg2^viii^	111.37 (6)	Mg1^xxii^—O3—Zn1^xxii^	0.0
Mg1^ii^—Mg2—O3^ix^	147.6 (2)	Mg1^xxii^—O3—V1^xii^	145.2 (5)
Mg1^ii^—Mg2—O3^v^	38.2 (2)	Mg2^ix^—O3—Zn1^xxii^	94.3 (4)
Mg1^ii^—Mg2—O5^x^	105.2 (3)	Mg2^ix^—O3—V1^xii^	115.7 (4)
Mg1^ii^—Mg2—O5^iv^	42.0 (3)	Zn1^xxii^—O3—V1^xii^	145.2 (5)
Mg1^ii^—Mg2—O6^vi^	89.7 (3)	Mg1^xxii^—O4—Zn1^xxii^	0.0
Mg1^ii^—Mg2—O6^xi^	123.3 (3)	Mg1^xxii^—O4—V2	154.6 (5)
Mg1^vii^—Mg2—Mg2^viii^	111.37 (6)	Zn1^xxii^—O4—V2	154.6 (5)
Mg1^vii^—Mg2—O3^ix^	38.2 (2)	Mg1^v^—O5—Mg2^xxiii^	101.1 (5)
Mg1^vii^—Mg2—O3^v^	147.6 (2)	Mg1^v^—O5—Zn1^v^	0.0
Mg1^vii^—Mg2—O5^x^	42.0 (3)	Mg1^v^—O5—Zn2^xxiii^	101.1 (5)
Mg1^vii^—Mg2—O5^iv^	105.2 (3)	Mg1^v^—O5—V3^xvii^	116.7 (5)
Mg1^vii^—Mg2—O6^vi^	123.3 (3)	Mg2^xxiii^—O5—Zn1^v^	101.1 (5)
Mg1^vii^—Mg2—O6^xi^	89.7 (3)	Mg2^xxiii^—O5—Zn2^xxiii^	0.0
Mg2^viii^—Mg2—O3^ix^	85.4 (2)	Mg2^xxiii^—O5—V3^xvii^	136.5 (5)
Mg2^viii^—Mg2—O3^v^	85.4 (2)	Zn1^v^—O5—Zn2^xxiii^	101.1 (5)
Mg2^viii^—Mg2—O5^x^	131.3 (3)	Zn1^v^—O5—V3^xvii^	116.7 (5)
Mg2^viii^—Mg2—O5^iv^	131.3 (3)	Zn2^xxiii^—O5—V3^xvii^	136.5 (5)
Mg2^viii^—Mg2—O6^vi^	41.7 (3)	Mg2^xxiv^—O6—Mg2^ix^	96.5 (6)
Mg2^viii^—Mg2—O6^xi^	41.7 (3)	Mg2^xxiv^—O6—Zn2^xxiv^	0.0
O3^ix^—Mg2—O3^v^	170.8 (4)	Mg2^xxiv^—O6—Zn2^ix^	96.5 (6)
O3^ix^—Mg2—O5^x^	80.2 (3)	Mg2^xxiv^—O6—V3^xvii^	131.6 (3)
O3^ix^—Mg2—O5^iv^	106.1 (4)	Mg2^ix^—O6—Zn2^xxiv^	96.5 (6)
O3^ix^—Mg2—O6^vi^	85.2 (4)	Mg2^ix^—O6—Zn2^ix^	0.0
O3^ix^—Mg2—O6^xi^	87.9 (4)	Mg2^ix^—O6—V3^xvii^	131.6 (3)
O3^v^—Mg2—O5^x^	106.1 (4)	Zn2^xxiv^—O6—Zn2^ix^	96.5 (6)
O3^v^—Mg2—O5^iv^	80.2 (3)	Zn2^xxiv^—O6—V3^xvii^	131.6 (3)
O3^v^—Mg2—O6^vi^	87.9 (4)	Zn2^ix^—O6—V3^xvii^	131.6 (3)
O3^v^—Mg2—O6^xi^	85.2 (4)	Mg1^xxiv^—O7—Mg1^v^	96.4 (6)
O5^x^—Mg2—O5^iv^	97.3 (5)	Mg1^xxiv^—O7—Zn1^xxiv^	0.0
O5^x^—Mg2—O6^vi^	164.7 (4)	Mg1^xxiv^—O7—Zn1^v^	96.4 (6)
O5^x^—Mg2—O6^xi^	91.2 (4)	Mg1^xxiv^—O7—V2^xxv^	109.8 (4)
O5^iv^—Mg2—O6^vi^	91.2 (4)	Mg1^xxiv^—O7—V2^xvii^	120.3 (4)
O5^iv^—Mg2—O6^xi^	164.7 (4)	Mg1^v^—O7—Zn1^xxiv^	96.4 (6)
O6^vi^—Mg2—O6^xi^	83.5 (6)	Mg1^v^—O7—Zn1^v^	0.0
O3^iv^—Zn1—O4^iv^	93.5 (3)	Mg1^v^—O7—V2^xxv^	109.8 (4)
O3^iv^—Zn1—O5^v^	84.3 (3)	Mg1^v^—O7—V2^xvii^	120.3 (4)
O3^iv^—Zn1—O7^vi^	103.6 (5)	Zn1^xxiv^—O7—Zn1^v^	96.4 (6)
O3^iv^—Zn1—O8^vi^	167.9 (4)	Zn1^xxiv^—O7—V2^xxv^	109.8 (4)
O4^iv^—Zn1—O5^v^	114.4 (4)	Zn1^xxiv^—O7—V2^xvii^	120.3 (4)
O4^iv^—Zn1—O7^vi^	128.8 (4)	Zn1^v^—O7—V2^xxv^	109.8 (4)
O4^iv^—Zn1—O8^vi^	94.1 (4)	Zn1^v^—O7—V2^xvii^	120.3 (4)
O5^v^—Zn1—O7^vi^	115.1 (4)	V2^xxv^—O7—V2^xvii^	100.1 (7)
O5^v^—Zn1—O8^vi^	84.0 (5)	Mg1^xxiv^—O8—Mg1^v^	94.1 (7)
O7^vi^—Zn1—O8^vi^	78.8 (4)	Mg1^xxiv^—O8—Zn1^xxiv^	0.0
O5^x^—Zn2—O5^iv^	97.3 (5)	Mg1^xxiv^—O8—Zn1^v^	94.1 (7)
O5^x^—Zn2—O6^vi^	164.7 (4)	Mg1^xxiv^—O8—V1	132.7 (3)
O5^x^—Zn2—O6^xi^	91.2 (4)	Mg1^v^—O8—Zn1^xxiv^	94.1 (7)
O5^iv^—Zn2—O6^vi^	91.2 (4)	Mg1^v^—O8—Zn1^v^	0.0
O5^iv^—Zn2—O6^xi^	164.7 (4)	Mg1^v^—O8—V1	132.7 (3)
O6^vi^—Zn2—O6^xi^	83.5 (6)	Zn1^xxiv^—O8—Zn1^v^	94.1 (7)
O2—V1—O3^xii^	104.6 (3)	Zn1^xxiv^—O8—V1	132.7 (3)
O2—V1—O3^xiii^	104.6 (3)	Zn1^v^—O8—V1	132.7 (3)
O2—V1—O8	104.2 (5)		

Symmetry codes: (i) *x*, −*y*+2, *z*; (ii) −*x*+1/2, −*y*+3/2, −*z*+1; (iii) −*x*+1, *y*+1, −*z*+1; (iv) −*x*+1/2, *y*+1/2, −*z*+1; (v) *x*, −*y*+1, *z*; (vi) *x*, *y*+1, *z*; (vii) *x*−1/2, −*y*+3/2, *z*; (viii) −*x*, −*y*+2, −*z*+1; (ix) −*x*, −*y*+1, −*z*+1; (x) *x*−1/2, *y*+1/2, *z*; (xi) −*x*, *y*+1, −*z*+1; (xii) −*x*, *y*, −*z*+1; (xiii) −*x*, −*y*, −*z*+1; (xiv) −*x*+1, *y*, −*z*+2; (xv) *x*, −*y*, *z*; (xvi) *x*, *y*, *z*+1; (xvii) −*x*+1, *y*, −*z*+1; (xviii) −*x*+1, *y*−1, −*z*+1; (xix) −*x*+1, −*y*+1, −*z*+1; (xx) −*x*+1, −*y*, −*z*+1; (xxi) −*x*, *y*, −*z*; (xxii) −*x*+1/2, *y*−1/2, −*z*+1; (xxiii) *x*+1/2, *y*−1/2, *z*; (xxiv) *x*, *y*−1, *z*; (xxv) *x*, *y*, *z*−1.
